# BMI and Fracture Risk in Older Men: The Osteoporotic Fractures in Men Study (MrOS)

**DOI:** 10.1002/jbmr.235

**Published:** 2010-09-02

**Authors:** Carrie M Nielson, Lynn M Marshall, Annette L Adams, Erin S LeBlanc, Peggy M Cawthon, Kristine Ensrud, Marcia L Stefanick, Elizabeth Barrett-Connor, Eric S Orwoll

**Affiliations:** 1 School of Medicine, Oregon Health & Science UniversityPortland, OR, USA; 2 Kaiser Permanente Southern CaliforniaPasadena, CA, USA; 3 Kaiser Permanente Northwest, Center for Health ResearchPortland, OR, USA; 4 California Pacific Medical CenterSan Francisco, CA, USA; 5 Veterans Affairs Medical Center and University of MinnesotaMinneapolis, MN, USA; 6 Stanford UniversityStanford, CA, USA; 7 Department of Family and Preventive Medicine, University of CaliforniaSan Diego, La Jolla, CA, USA

**Keywords:** FRACTURE, BMI, MEN, INCIDENCE, OBESITY

## Abstract

Low body mass index (BMI) is a risk factor for fracture, but little is known about the association between high BMI and fracture risk. We evaluated the association between BMI and fracture in the Osteoporotic Fractures in Men Study (MrOS), a cohort of 5995 US men 65 years of age and older. Standardized measures included weight, height, and hip bone mineral density (BMD) by dual-energy X-ray absorptiometry (DXA); medical history; lifestyle; and physical performance. Only 6 men (0.1%) were underweight (<18.5 kg/m^2^); therefore, men in this category were excluded. Also, 27% of men had normal BMI (18.5 to 24.9 kg/m^2^), 52% were overweight (25 to 29.9 kg/m^2^), 18% were obese I (30 to 34.9 kg/m^2^), and 3% were obese II (35 to 39.9 kg/m^2^). Overall, nonspine fracture incidence was 16.1 per 1000 person-years, and hip fracture incidence was 3.1 per 1000 person-years. In age-, race-, and BMD-adjusted models, compared with normal weight, the hazard ratio (HR) for nonspine fracture was 1.04 [95% confidence interval (CI) 0.87–1.25] for overweight, 1.29 (95% CI 1.00–1.67) for obese I, and 1.94 (95% CI 1.25–3.02) for obese II. Associations were weaker and not statistically significant after adjustment for mobility limitations and walking pace (HR = 1.02, 95% CI 0.84–1.23, for overweight; HR = 1.12, 95% CI 0.86–1.46, for obese I, and HR = 1.44, 95% CI 0.90–2.28, for obese II). Obesity is common among older men, and when BMD is held constant, it is associated with an increased risk of fracture. This association is at least partially explained by worse physical function in obese men. © 2011 American Society for Bone and Mineral Research.

## Introduction

Osteoporotic fractures among men are a major public health problem in the United States and worldwide.(1) Identifying modifiable risk factors for fractures in men is essential for fracture prevention. Prospective studies, including one from the Osteoporotic Fractures in Men Study (MrOS) cohort, have established that low hip bone mineral density (BMD) is an independent risk factor for fracture among older men.(2) However, the nonskeletal determinants of fracture risk among older men are not well understood. Because 30% of US adults age 60 or older are obese,(3) understanding the role of body mass index (BMI) in fracture risk is of particular relevance. Underweight has consistently been reported as a risk factor for fracture when compared to normal weight(4); however, the effects of overweight and obesity are unclear.

Although the adverse effects of adiposity on bone metabolism and bone density have been recently emphasized (reviewed in ref. 5), there is little understanding of body size and composition related to fracture risk among men. In particular, the influence of increased BMI and adiposity on fracture risk is unclear, and results of existing studies are equivocal. Some reports in men suggest an increased risk of fracture among men with greater adiposity(6); however, other studies show either no association,(7,8) an increased risk of fracture for those with low BMI,(4,9–11) or associations that differ by fracture site.(12) In another study, the risk of hip and wrist fracture were twofold greater among men with the greatest waist circumference compared with men with the lowest, indicating the potential relevance of body fat distribution to fracture risk in men.(13) Among postmenopausal women presenting with a low-trauma fracture, the prevalence of obesity was about 27%, although the majority had normal BMD.(14) Clarifying the relationship between adiposity and fracture risk is essential for identifying men at risk for fracture and devising appropriate preventive interventions.

We conducted a large prospective cohort study to determine risk factors for fracture among men aged 65 years and older. Participants in MrOS attended clinical examinations, at which height and weight were measured to determine BMI, and BMD was measured with dual-energy X-ray absorptiometry (DXA). The objectives of the current analysis were to determine the associations between BMI and fracture risk in aging men before and after adjustment for BMD and other potential confounders.

## Materials and Methods

### Study population

MrOS enrolled 5995 participants from March 2000 through April 2002. Recruitment occurred at six US clinical centers (Birmingham, AL, Minneapolis, MN, Palo Alto, CA, Pittsburgh, PA, Portland, OR, and San Diego, CA) and was accomplished primarily through mass mailings targeted to age-eligible men. Eligible participants were community-dwelling men who were at least 65 years of age, able to walk without assistance from another person, and had not had bilateral hip replacements (in order to obtain a hip BMD measure). Details of the MrOS study design and recruitment have been published elsewhere.(15,16) Written informed consent was obtained from all participants, and the institutional review board at each study site approved the study.

### Clinic visits

Participants completed self-administered questionnaires and attended a baseline and follow-up clinic visit, when anthropometric and skeletal measures were obtained, including the height (cm) and weight (kg) from which BMI was computed (kg/m^2^). World Health Organization categories of BMI were used: Underweight was less than 18.5 kg/m^2^, normal BMI was 18.5 to 24.9 kg/m^2^, overweight was 25 to 29.9 kg/m^2^, obese I was 30 to 34.9 kg/m^2^, and obese II was 35 to 39.9 kg/m^2^. Only six men (0.1%) were underweight, and 29 had BMI ≥ 40 kg/m^2^; therefore, the men in these categories were excluded from the analyses. Demographic, medical history, lifestyle, and lifestyle factors also were obtained from standardized questionnaires and measures at the clinic visits.(16) History of falls and mobility limitations (difficulty walking two to three blocks outside on level ground or climbing up 10 steps) were ascertained by questionnaire. Dietary calcium and vitamin D were assessed using a modified Block Food Frequency Questionnaire.(17) Physical activity was assessed with the Physical Activity Scale for the Elderly (PASE).(18)

The physical performance measures were chair stand time, narrow walk pace, leg power, and grip strength. For the chair stand, participants were asked to stand from a chair without using their arms; those who could not perform a single chair stand were classified as “unable” to complete the test. All men who were able to complete the single chair stand were asked to perform the repeated chair stand test. The ability and time required to complete five chair stands without using the arms were recorded. Participants who were unable to do five chair stands, used their arms at any time during the test, or refused to do the repeated chair stand test also were classified as “unable.” Participants were asked to complete a narrow walk trial over a course 20 cm wide and 6 m long, and the time (seconds) to complete the trial was recorded. Inability to complete a trial (eg, stepping outside the line) was recorded. Leg power was measured using the Nottingham Power Rig,(19) and the maximum of five trials was used. Average grip strength (kg) from a handheld dynameter (Jamar Hydraulic Hand Dynamometer, Sammons Preston, Inc., Bolingbrook, IL, USA) was used in analysis.

Participants also reported cigarette smoking (current, past, or never) and alcohol use (drinks per week). Participants were asked to bring in all prescription medications used within the last 30 days. A computerized dictionary, based on the original Established Populations for Epidemiologic Studies of the Elderly (EPESE) coding system,(20) was used to categorize medications. All recorded prescription medications were stored in an electronic medications inventory database (San Francisco Coordinating Center, San Francisco, CA, USA). Each medication was matched to its ingredient(s) based on the Iowa Drug Information Service (IDIS) Drug Vocabulary (College of Pharmacy, University of Iowa, Iowa City, IA, USA).

### Bone density measurements

BMD (g/cm^2^) was measured using fan-beam DXA (QDR 4500W, Hologic Inc., Waltham, MA, USA), as described previously.(16) A central quality-control lab, certification of DXA operators, and standardized procedures for scanning were used to ensure reproducibility of DXA measurements. Regular scans of Hologic hip, linearity, and whole-body phantoms were made at baseline and throughout the enrollment period at all study sites to verify that machine performance remained stable. Cross-calibration studies performed among the six clinical centers prior to the baseline MrOS visit found no linear differences among scanners.

The variability across clinics was within acceptable limits, and cross-calibration correction factors were not required. Validity of these measures by DXA for the assessment of body composition in the elderly has been determined.(21,22) Complete baseline height, weight, and DXA measures were available for 5953 participants (99.3% of the total MrOS cohort).

### Follow-up and fracture ascertainment

Fracture events were reported by participants at 4-month intervals on brief mailed questionnaires. Subsequently, study physicians centrally adjudicated reported fractures from medical records. For this analysis, fracture types were defined as all nonspine fractures, upper extremity fractures (ie, arm, shoulder, or wrist), lower extremity fractures (ie, leg or ankle), and hip fractures. Because exclusion of traumatic fractures has been reported to underestimate the contribution of osteoporosis to fractures in women and men,(23,24) such fractures were not excluded in the current analysis. During follow-up, next of kin were contacted for men with unreturned questionnaires who could not be reached by telephone. Deaths were confirmed with death certificates.

### Statistical analysis

Differences in baseline characteristics according to BMI category were tested using ANOVA or chi-square tests. Pearson's correlation coefficient (*r*) between total-hip BMD and BMI was calculated, and the distributions of BMI across BMD quintiles were plotted. Fracture incidence rates per 1000 person-years were calculated for each BMI category. Incidence-rate ratios were used to test differences from the normal BMI category. The hazard ratio was used to estimate relative risk for each BMI category compared with normal BMI for fracture outcomes including nonspine fracture and fractures of the hip, upper extremities, and lower extremities.

In Cox proportional hazards regression models, independent variables were examined as time-varying covariates to allow for all values, including BMI, obtained at a follow-up visit to be updated in the analysis. Restricted cubic spline models were used to visualize the shape of the association between BMI and fracture incidence, both before and after BMD adjustment, and to test for nonlinearity in the associations.(25) No nonlinear effects were detected. For the Cox proportional hazards models, we fit three models, in which we evaluated increasing numbers of covariates. The first was adjusted for age and race. The second was further adjusted for total-hip BMD to examine the association of BMI with fracture while holding BMD constant. Third, we evaluated several potential confounding factors (including those listed in Table 1) and retained them in the final models if they altered the association between nonspine fracture and any of the BMI categories by 5% or more. The final multivariable model included adjustments for age, race, total-hip BMD, baseline history of fractures, self-reported mobility limitation, and narrow walk pace. Diabetes was specifically considered as a potential confounder, but adjustment for diabetes status did not alter results or conclusions.

**Table 1:** Selected Baseline Characteristics by Body Mass Index Category, the Osteoporotic Fractures in Men (MrOS) Study

		Body mass index category				
			
Characteristic	All men (*n* = 5918)	Normal (18.5–24.9 kg/m^2^) (*n* = 1628)	Overweight (25–29.9 kg/m^2^) (*n* = 3049)	Obese I (30–34.9 kg/m^2^) (*n* = 1034	Obese II (35–39.9 kg/m^2^) (*n* = 207)	*p* Value
Body mass index, range in kg/m^2^	18.5–39.9	17.2–24.3	24.3–26.1	26.1–27.9	27.9–30.2	
Body mass index, kg/m^2^	27.3 (3.6)	22.7 (1.3)	25.2 (0.5)	26.9 (0.5)	28.9 (0.7)	<.001
Total-hip BMD, range in g/cm^2^	0.31–1.76	0.31–1.43	0.45–1.73	0.53–1.43	0.63–1.49	
Total-hip BMD, g/cm^2^	0.96 (0.14)	0.90 (0.13)	0.96 (0.13)	1.02 (0.14)	1.06 (0.13)	<.001
Age, years	73.6 (5.9)	75.0 (6.4)	74.4 (6.0)	73.7 (5.9)	72.9 (5.3)	<.001
Race, white, *n* (%)	5295 (89.5)	1445 (88.8)	2742 (89.9)	925 (89.5)	183 (88.4)	.61
History of falls in the past year, *n* (%)	1248 (21.1)	365 (22.4)	604 (19.8)	224 (21.7)	55 (26.6)	.03
History of fractures, *n* (%)	3277 (55.4)	867 (53.3)	1701 (55.8)	580 (56.1)	129 (62.3)	.06
Mobility limitations, *n* (%)	804 (13.6)	157 (9.7)	372 (12.2)	210 (20.4)	65 (31.4)	<.001
PASE score	147 (68)	149 (70)	148 (67)	145 (67)	124 (67)	<.001
Grip strength, kg^a^	38.1 (9.1)	36.7 (8.8)	38.4 (9.1)	39.1 (8.9)	38.3 (10.2)	<.001
Unable, *n* (%)	101 (1.7)	32 (2.0)	49 (1.6)	12 (1.2)	8 (3.9)	.04
Narrow walk pace, m/s^a^	1.03 (0.43)	1.04 (0.42)	1.05 (0.42)	0.98 (0.43)	0.85 (0.44)	<.001
Unable, *n* (%)	732 (12.5)	192 (11.9)	346 (11.5)	152 (14.9)	42 (20.9)	<.001
Overall health, *n* (%)						
Excellent/good/fair	5826 (98.5)	1605 (98.7)	3012 (98.8)	1015 (98.2)	194 (93.7)	
Poor/very poor	91 (1.4)	22 (1.4)	37 (1.2)	19 (1.8)	13 (6.3)	<.001
Medical history, *n* (%)						
Diabetes	642 (10.9)	109 (6.7)	308 (10.1)	169 (16.3)	56 (27.1)	<.001
Osteoporosis	206 (3.5)	70 (4.3)	104 (3.4)	28 (2.7)	4 (1.9)	.09
Arthritis	2799 (47.3)	665 (40.9)	1423 (46.7)	578 (55.9)	133 (64.3)	<.001
Medication use, *n* (%)						
Loop diuretic	294 (5.2)	49 (3.1)	133 (4.6)	82 (8.2)	30 (14.8)	<.001
Thiazide diuretic	711 (12.5)	149 (9.5)	364 (12.5)	159 (16.0)	39 (19.2)	<.001
Beta blocker	1414 (27.2)	331 (23.0)	713 (26.6)	309 (34.6)	61 (34.7)	<.001

*Note:* Results are mean (SD) unless otherwise noted. PASE = physical activity score for the elderly

a Participants who were unable to complete a test were coded with the lowest or slowest reported value. *p* Value is for trend or chi-square test.

Incidence rate ratios and 95% confidence intervals (CIs) were calculated in Stata 9.2 (Stata Corporation, Inc., College Station, TX), and all other analyses were conducted using SAS Version 9.1 (SAS Institute, Inc., Cary, NC, USA).

## Results

**Fig. 1:**
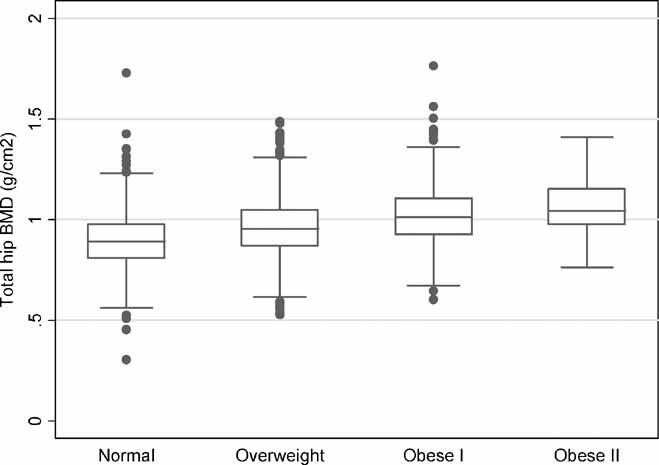
Distribution of BMD by BMI category, the Osteoporotic Fractures in Men (MrOS) study.

The mean BMI was 27 kg/m^2^, which was similar to that of US men in this age group.(26) Most men in our study (72%) were overweight or obese. Only six men were underweight, and 29 had a BMI of 40 kg/m^2^ or greater (and therefore were excluded from our analyses of the association between BMI and fracture outcomes). Age, BMD, mobility limitations, and exercise frequency varied significantly by BMI category (Table 1). Men in higher BMI categories scored lower on physical performance measures and had worse health histories and more medication use despite being younger, on average, than those in the lower BMI categories. A greater proportion of men in the obese II category reported a history of falls at baseline (26.6%) than did men in lower BMI categories (19.8% to 22.4%). The age-adjusted correlation between total-hip BMD and BMI was moderate (Pearson's *r* = 0.35, *p* < .0001). Each BMI category encompassed a wide range of BMD values (Fig. 1). Mean BMD in heavier men was higher than in lighter men, but many overweight and obese men had BMD in the same range as normal weight men (Table 1).

During a mean of 7.0 ± 1.6 years of follow-up, 634 confirmed nonspine fractures occurred; 126 were hip fractures. The incidence rate of nonspine fracture was 16.1 per 1000 person-years and of hip fracture was 3.1 per 1000 person-years. Just as most cohort members were overweight or obese, most nonspine fracture cases (68%) and hip fracture cases (62%) occurred in overweight or obese men (Table 2). Fracture incidence was higher in the normal-weight and most obese II men than in the overweight and obese I men for all nonspine fractures, hip fractures, and upper extremity fractures; however, differences were not statistically significant.

**Table 2:** Incident Fractures, by Location, in Relation to Body Mass Index Among Older Men, the Osteoporotic Fractures in Men (MrOS) Study

	Normal (18.5–24.9 kg/m^2^) (*n* = 1628)	Overweight (25–29.9 kg/m^2^) (*n* = 3049)	Obese I (30–34.9 kg/m^2^) (*n* = 1034)	Obese II (35–39.9 kg/m^2^) (*n* = 207)
Nonspine fractures				
No. fractures/person-years	202/10,468	309/20,480	97/6878	24/1356
Incidence rate^a^	19.3	15.1	14.1	17.7
Adjusted hazard ratios				
Age and race	Ref	0.81 (0.68–0.97)	0.83 (0.65–1.05)	1.08 (0.71–1.66)
Age, race, and BMD	Ref	1.04 (0.87–1.25)	1.29 (1.00–1.67)	1.94 (1.25–3.02)
Full model^b^	Ref	1.02 (0.84–1.23)	1.12 (0.86–1.46)	1.44 (0.90–2.28)
Hip fractures				
No. fractures/person-years	48/11,021	61/21,354	12/7157	5/1403
Incidence rate^a^	4.4	2.9	1.7	3.6
Adjusted hazard ratios				
Age and race	Ref	0.72 (0.49–1.05)	0.65 (0.35–1.19)	1.17 (0.42–3.29)
Age, race, and BMD	Ref	1.25 (0.82–1.89)	1.76 (0.92–3.34)	5.04 (1.74–14.6)
Full model^b^	Ref	1.24 (0.78–1.97)	1.34 (0.66–2.74)	3.17 (1.04–9.71)
Upper extremity fractures				
No. fractures/person-years	53/10,937	84/21,203	21/7105	6/1401
Incidence rate^a^	4.8	4.0	3.0	4.3
Adjusted hazard ratios				
Age and race	Ref	0.92 (0.65–1.31)	0.77 (0.46–1.28)	1.17 (0.50–2.76)
Age, race, and BMD	Ref	1.25 (0.87–1.80)	1.35 (0.78–2.32)	2.43 (1.00–5.90)
Full model^b^	Ref	1.33 (0.92–1.94)	1.27 (0.73–2.21)	1.85 (0.70–4.92)
Lower extremity fractures				
No. fractures/person-years	22/10,986	61/21,172	24/7070	6/1398
Incidence rate^a^	2.0	2.9	3.4	4.3
Adjusted hazard ratios				
Age and race	Ref	1.34 (0.84–2.14)	1.56 (0.89–2.74)	1.71 (0.66–4.44)
Age, race, and BMD	Ref	1.59 (0.99–2.56)	2.15 (1.20–3.87)	2.57 (0.97–6.79)
Full model^b^	Ref	1.54 (0.94–2.53)	1.84 (0.98–3.46)	1.82 (0.66–5.00)

a Per 1000 person-years.

b Adjusted for age, race, total-hip BMD, baseline history of fracture, self-reported mobility limitation, and narrow walk pace. Upper extremity fractures include arm, shoulder, and wrist. Lower extremity fractures include leg and ankle.

In models adjusted for age and race, overweight was associated with a 19% lower risk of nonspine fracture risk (Table 2) compared with normal weight, whereas associations with the highest BMI categories were weaker and not statistically significant. However, after adjustment for total-hip BMD, the risk of all nonspine fracture was 29% greater in obese I and nearly twofold greater obese II men compared with men of normal weight. Similarly, the risk of hip and upper and lower extremity fractures was not different between BMI groups prior to adjustment for BMI but was higher in obese men after BMD adjustment (Table 2).

We sought to identify factors that might be related to the increased risk associated with obesity after BMD adjustment. Mobility limitations—difficulty climbing 10 steps or walking two to three blocks—were reported by 31.4% of men in the obese II category and 9.7% of men with normal BMI. When this variable was included in age-, race-, and BMD-adjusted models of nonspine fracture, the hazard ratio for the obese II category was attenuated. Additional adjustment for narrow walk pace and baseline history of fracture further attenuated the nonspine fracture association. No medical condition, medication use, or history of falls variable altered the associations by 5% or more.

## Discussion

While the risk of osteoporotic fractures, especially hip fractures, has consistently been reported to be higher in those with low BMI, fracture risk in overweight and obese individuals has not been well characterized. In this large prospective study of older men who were normal weight or heavier, most hip (62%) and nonspine fractures (68%) occurred in overweight or obese men, who represented 72% of the cohort. We also found that obesity was associated with an increased risk of hip and other fractures after adjustment for BMD, but this association appeared to be due at least in part to confounding by deficits in physical performance. Similar deficits have been linked to fall rates(27,28) and might mediate the obesity-fracture relationship. However, adjustment for a history of falls in the previous year, which has been consistently predictive of incident falls,(28,29) did not alter associations. In light of our finding that many fractures occur in men of higher BMI, public health and clinical strategies must be developed to identify overweight and obese individuals at greatest risk. Moreover, since obesity appears to impart particular risk for fracture after BMD adjustment, the mechanisms that are responsible must be determined.

Whereas some previous analyses have noted a tendency for increased weight to be associated with higher fracture risk,(6,30,31) the MrOS study design made it possible to examine fracture and BMI associations in ranges more typical of in men in the United States Very few MrOS men were in the clinical underweight category (<18.5 kg/m^2^, *n* = 6), and the majority of participants were overweight or obese, which is reflective of US men in this age group.(26) Many other studies that previously examined the association between fracture and BMI have been characterized by relatively lower weights, and the mean baseline BMI in MrOS (27.4 kg/m^2^) is higher than the mean for men in a recent meta-analysis of seven studies of BMI and fracture(4) that focused on the relationship of low weight and fracture risk. Our findings are not incompatible with studies that have reported a higher risk of fracture for men with low BMI than for men with a normal BMI. In fact, in the meta-analysis of BMI and fracture in men, the association was observed to be nonlinear, with greatest risk in low BMI and no additional reduction of risk among men with greater than normal BMI.(4) Whereas the importance of low weight as a risk factor for fracture should not be ignored, the proportion of men 70 to 79 years of age in the United States who are underweight is small [<5% from the National Health and Nutrition Examination Survey (NHANES)], and more than half are overweight or obese.(26) The chronic disease risks associated with obesity have long been recognized, and our results support the conclusion that clinical and public health messages for obesity prevention should be compatible with fracture-prevention messages.

BMD is moderately positively correlated with BMI, and low BMD has been considered one of the major causes of the increased risk of fracture in those with low weight. Similarly, higher BMD in larger people is posited to mitigate fracture risk. On the other hand, the correlation between BMI and BMD is moderate, many overweight and obese individuals have relatively low BMD, and bone strength may not increase in proportion to increases in total or fat mass.(31) Moreover, fat gain has been associated with higher rates of BMD loss,(32) and visceral fat in particular has been shown to be negatively correlated with bone structure and strength.(33) Thus increased adiposity may have deleterious effects on bone strength that could be important in determining fracture risk.

In addition to the possible adverse effects of fat on bone strength, our results suggest that obesity also imparts a considerably higher risk of hip and other fractures after BMD adjustment. Some data suggest that obesity may impair physical function and increase the risk of falls.(34,35) On the other hand, in women, increased adiposity may protect against hip fracture by reducing the force exerted on bone in a fall.(36) However, we reported previously that soft tissue thickness around the hip did not protect against fracture in men.(37) This observation suggests the possibility of gender differences in the relation of excess adiposity to fracture risk.

In obese men, disruption of the hypothalamic-pituitary axis, including androgen deficiency, has been reported, and higher BMI is be associated with lower serum vitamin D levels.(38,39) These are plausible mediators of an obesity-fracture association; however, only a subset of men in the MrOS cohort had these measures available. Furthermore, we have reported previously that low testosterone was not associated with increased fracture risk in the MrOS cohort.(40) We also reported an increased risk of hip fracture in men with low serum vitamin D that was attenuated with adjustment for BMD.(41) Further investigation into neuroendocrine alterations in obesity as they relate to fracture risk may provide additional insight.

The limitations of this study include the use of a relatively healthy volunteer cohort in which there were few underweight men, a group that is known to be at particularly high risk of fracture. While this prevented us from examining the risk of underweight in our analyses, in view of the weight distribution in the US population of older men, we were most interested in the effects of overweight and obesity. Also, 90% of men were non-Hispanic whites, which may limit generalizability. Different associations with body composition and fracture between women and men have consistently been reported, and our conclusions cannot be applied to fracture risk in women. Weight loss has been shown to be associated with BMD loss(42) and increased hip fracture risk,(43) but we lacked detailed information about weight and weight change prior to enrollment. As in any association study, there may be unmeasured confounders that explain the effect of BMI on both BMD and fracture. Finally, although we examined associations with fractures in different regions of the body, we lacked power to further dissect fracture sites. For example, associations may differ by whether the hip fracture is trochanteric(44) or by whether an arm fracture occurs in the forearm versus the proximal humerus.(9)

In summary, in this large cohort of older men, most of whom had a BMI above normal, most hip and other nonspine fractures occurred in those who were overweight or obese, which reflected the distribution of BMI in the cohort. In addition, obesity was an important contributor to the risk of fracture, including hip fracture, among men with similar BMD. The combination of hip fracture and obesity, both of which adversely affect physical function,(45–47) may be particularly likely to lead to disability or institutionalization.(45,48) The interplay of BMI, BMD, physical performance, and fracture requires additional study in the context of a growing population of overweight and obese older men in the United States.
